# Association of the Neighborhood Built Environment With Incident and Prevalent Depression in the Rural South

**DOI:** 10.5888/pcd18.200605

**Published:** 2021-07-08

**Authors:** Christopher E. Anderson, Stephanie T. Broyles, Maeve E. Wallace, Lydia A. Bazzano, Jeanette Gustat

**Affiliations:** 1 Department of Epidemiology, School of Public Health and Tropical Medicine, Tulane University, New Orleans, Louisiana; 2 Pennington Biomedical Research Center, Louisiana State University, Baton Rouge, Louisiana; 3 Department of Social, Behavioral, and Population Sciences, School of Public Health and Tropical Medicine, Tulane University, New Orleans, Louisiana

## Abstract

**Introduction:**

A neighborhood’s built environment is associated with physical activity among its residents, and physical activity is associated with depression. Our study aimed to determine whether the built environment was associated with depression among residents of the rural South and whether observed associations were mediated by physical activity.

**Methods:**

We selected 2,000 participants from the Bogalusa Heart Study who had a valid residential address, self-reported physical activity (minutes/week), and a complete Center for Epidemiologic Study–Depression (CES-D) scale assessment from 1 or more study visits between 1998 and 2013. We assessed the built environment with the Rural Active Living Assessment street segment audit tool and developed built environment scores. The association between built environment scores and depression (CES-D ≥16) in geographic buffers of various radii were evaluated by using modified Poisson regression, and mediation by physical activity was evaluated with mixed-effects models.

**Results:**

Depression was observed in 37% of study participants at the first study visit. One-point higher physical security and aesthetic scores for the street segment of residence were associated with 1.07 times higher (95% CI, 1.02–1.11) and 0.96 times lower (95% CI, 0.92–1.00) baseline depression prevalence. One-point higher destination scores (ie, more commercial and civic facilities) in radius buffers of 0.25 miles or more were associated with 1.06 times (95% CI, 1.00–1.13) the risk of depression during follow-up. Neighborhood poverty (defined as percentage of residents with incomes below the federal poverty level and dichotomized at 28.3%) modified cross-sectional and longitudinal associations. Associations were not mediated by physical activity.

**Conclusion:**

The built environment was associated with prevalence and risk of depression, and associations were stronger in high-poverty neighborhoods. Built environment improvements to promote physical activity should take neighborhood context into consideration to minimize negative side effects on mental health in high-poverty communities.

**Summary**

**What is already known on this topic?**

Features of the built environment are associated with physical activity in urban and rural communities and with depression in urban communities.

**What is added by this report?**

Features of the built environment, including aesthetics, destinations, and security, were associated with depression in a rural population in Louisiana, and these associations were not mediated by physical activity.

**What are the implications for public health practice?**

Improvements in the built environment that promote physical activity among rural populations should take neighborhood context into consideration to minimize negative side effects on mental health.

## Introduction

Depression is among the leading causes of years lived with disability worldwide (1). Nearly 10% of US adults experience depression, a substantial public health problem (2). Residents of rural areas are less likely to engage in sufficient physical activity than urban residents (3), contributing to elevated prevalence of chronic disease and health disparities among rural populations (4). However, rural populations experience fewer mental health disorders than intermediate-size urban areas (5).

Depression is associated with disorder and violence in the neighborhood environment and less consistently with structural features of the built environment (6). Most prior analyses of the relationship between depression and the built environment were of urban areas and were cross-sectional, with little consideration of spatial scale (6,7). Built environments may influence depressive symptoms as a neighborhood stressor (8) or along pathways mediated by behaviors (eg, physical activity) that result from the interactions of individuals with their environment (9). Built environment features that impede physical activity are more prevalent in rural locales than in urban ones. For example, rural residents may have greater distances to travel, roads with higher speed limits, and fewer pedestrian or cyclist safety features (10). Research has not determined whether depression among rural populations is associated with the built environment and whether it is mediated by physical activity.

We evaluated cross-sectional and longitudinal associations between structural features of the rural built environment and depressive symptoms among participants in the Bogalusa Heart Study. Previous research in this population identified significant associations between scores for features of the neighborhood built environment and physical activity (11). We hypothesized that higher scores (environments more conducive to physical activity) would be associated with lower baseline prevalence of depression and lower depression incidence and that these associations would be mediated by physical activity.

## Methods

The Bogalusa Heart Study is a longitudinal study of cardiovascular risk factors conducted in rural Washington Parish, Louisiana, that began in 1973 (12). Our cross-sectional analysis consisted of participants with a valid address of residence who had complete data on depressive symptoms and physical activity assessed in at least 1 study visit since 1998 (n = 2,000). Participants in the longitudinal analysis had more than 1 observation (range, 2–5 observations; mean, 2.55). Depressive symptoms were reported by study participants by using the Centers for Epidemiologic Studies–Depression (CES-D) scale, which has high validity in noninstitutionalized adult populations (13). Continuous CES-D scores were used in mixed models for the association of neighborhood environment with changes in severity of depressive symptoms, and CES-D was dichotomized (≥16, depressed; <16, not depressed) (14) for Poisson regression for cross-sectional (at the first CES-D assessment for each participant) and longitudinal associations of neighborhood environment with depression.

We audited built environment features of street segments of residence (n =1,340) by using Google Street View for each available image (n = 2,648) for all study participants (n = 2,000) by using the Rural Active Living Assessment (RALA) street segment audit tool (15). Built environment audits were merged to participant study data by date, with the most temporally proximate street segment image used for built environment exposure at each study visit. Reliability of built environment audits using Google Street View has been reported as high (16). Neighborhood scales were developed for all features assessed, and for features in domains of path, pedestrian safety, aesthetics, commercial and civic destinations, physical security, and land use. Reliability of the neighborhood scales, assessed with intraclass correlation coefficients for duplicate audits of 196 street segments, has previously been reported to be acceptable for all domains except physical security features (11).

### Covariates

Participants were characterized with anthropometric, demographic, socioeconomic, behavioral, and health covariates. Age, body mass index (BMI) (weight in kg/height in m^2^), and total physical activity were available as continuous variables. Self-reported data were dichotomized for education (≥high school degree, <than a high school degree), annual income (≥$15,000, <$15,000), marital status (married, unmarried), health insurance (yes, no), home ownership (yes, no), employment status (employed, unemployed), and alcohol consumption in the past 12 months (yes, no). Race was self-reported as White or Black. Smoking was self-reported and categorized (current, former, never). Neighborhood contextual variables were obtained for the census tract of residence from the American Community Survey 5-year estimates and the 2010 census (17). Variables for neighborhood poverty (the percentage of residents in a census tract living in a household with an income below the federal poverty level [FPL]) and population density (residents per square mile) were calculated and used as continuous variables and dichotomized at the sample mean for the evaluation of effect modification. High and low categories were defined for neighborhood population density (high density, ≥586 people/sq mi; low density, <586 people/sq mi) and percentage of residents living in neighborhood poverty (high poverty, ≥28.3%; low poverty, <28.3%).

### Analysis

We developed scales for built environment overall in 6 domains of street segment features identified a priori and refined following principal components analysis. This process has been reported in detail elsewhere (11). Briefly, scales were developed by creating 1 variable for features that were assessed across multiple RALA variables (eg, sidewalks, paths), with all variables coded so higher values indicated features that promote physical activity. We calculated variable means, and 1 point was added to a preliminary segment score for each variable for which the segment value exceeded the sample mean. The mean preliminary score was then calculated for segments with values above and below the sample mean for every variable, and these means were compared. We flagged variables for removal where the difference in mean preliminary score between segments above and below the sample mean was less than 1. A final segment score was calculated by adding 1 point for each unflagged variable where the segment value exceeded the sample mean for that variable. This process was done for all variables assessed (overall), and repeated within domains of features (ie, path, pedestrian safety, aesthetics, physical security, destinations, and land use). A built environment score of 0 indicated that the segment had no additional features that promote physical activity relative to the average street segment. This scoring process has been used in the development of walkability and playground indexes (18,19). The higher the score for a segment, the more features the segment contained thought to promote physical activity, with higher land use scores indicating more dense residential development, better-condition residences than the average street segment, and the absence of hills or other geographic or land development barriers to physical activity. Buffers — the area around each audited street segment — with radii of 0.25, 0.50, 1.00, and 1.50 miles were used to define neighborhood exposures. Overall neighborhood scores were calculated as the average, weighted by the inverse of the distance from the centroid, of street segments within these buffers.

We evaluated the association between neighborhood built environment scores and longitudinal change in severity of depressive symptoms by using a hierarchical, mixed-effects, linear growth model, including all study participants with 2 or more CES-D assessments (n = 1,006), with differences in the rate of change (slope) being the outcome of interest. Mixed-effects models were conducted for each built environment score, with an interaction between the score and age and random intercepts and coefficients for age for each subject nested in street segments and census tracts and adjusted for sex, education, smoking, neighborhood poverty, and population density. The average duration of follow-up for participants in the longitudinal analysis exceeded 10 years, so the slope differences were expressed as the number of CES-D points per 10-years of follow-up. The percentage of each association between built environment scores and depressive symptoms mediated by physical activity was evaluated in mixed effects linear growth regression models by using a product of coefficients method (20), and 95% CIs for the resultant percentage mediation were calculated with a specialized program (21) in SAS version 9.4 (SAS Institute).

The association between neighborhood built environment scores and depression was evaluated by using modified Poisson regression models with robust standard error estimation, which accommodated clustering in street segments and census tracts (22). We reported the cross-sectional association between the built environment and depression in the baseline examination as prevalence rate ratios (PRRs) and 95% CIs, adjusted for age, sex, education, smoking, neighborhood poverty, and population density. Models for the longitudinal association between neighborhood built environment and depression included all subjects with more than 1 depression assessment (n = 1,006), giving risk ratios (RR) and 95% CIs, adjusted for age at baseline, sex, baseline CES-D score, education, smoking, duration of time elapsed since baseline (follow-up duration), neighborhood poverty, and population density.

Neighborhood poverty and population density were identified a priori as potential effect modifiers. Modification of the effect of built environment scores on depression in Poisson regression models and depressive symptoms in mixed effects regression models was evaluated by interacting built environment scores with dichotomized percentage of neighborhood poverty or population density. All analyses were conducted by using SAS version 9.4. *P* values of <.05 were considered significant.

## Results

The mean values for subjects included in our cross-sectional analysis were on average age, 38.3; BMI, 29.8; CES-D score, 15.1; and reported weekly physical activity, 175.8 minutes (Table 1). A minority of the sample was male (43.1%), Black (33.6%), and had at least a high school education (41.9%). Most reported being a current or former smoker (55.7%), drinking alcohol in the past year (61.3%), having an annual household income at or above $15,000 (64.3%), owning their home (72.3%), being employed (79.8%), being married (53.2%), and being in good health (64.4%). Because subjects included in longitudinal analyses represent a subset of those in the cross-sectional analysis, they will not be described separately. Cross-sectional study participants who were older, more educated, and did not own their homes lived on street segments with significantly higher overall built environment scores, whereas only home ownership was significantly associated with lower overall built environment scores for the longitudinal sample. Cross-sectional analyses for depression at the baseline examination identified 740 depressed subjects (37.0% of 2,000 total subjects); longitudinal analyses for depression identified 568 depressed subjects and 438 nondepressed subjects at the end of follow-up (Table 2).

**Table 1:** Characteristics of Participants (N = 2,000) Included in Analyses Evaluating the Cross-Sectional and Longitudinal Associations of the Built Environment Around the Residence and Depression in a Rural Population, Bogalusa Heart Study, 1998–2013

Characteristic	Cross Sectional		Longitudinal	
	N = 2,000	*P* Value^a^	N = 1,006^b^	*P* Value^a^
Age, y, mean (SD)	38.27 (8.63)	.03	36.79 (4.96)	.05
BMI, mean (SD)	29.79 (8.03)	.18	29.63 (8.20)	.89
Follow-up duration, y, mean (SD)	0.00 (0.00)	1.00	10.68 (3.17)	.90
CES-D score^c^ at baseline, mean (SD)	15.08 (10.27)	.93	13.24 (9.84)	.75
Depressed at baseline, n (%)	740 (37.00)	.77	321 (31.91)	.46
Male, n (%)	862 (43.10)	.15	410 (41.46)	.77
Black, n (%)	672 (33.60)	.22	303 (30.64)	.79
≥High school education, n (%)	838 (41.90)	.002	548 (58.80)	.64
Household income ≥$15,000, n (%)	1,285 (64.25)	.87	718 (72.67)	.99
Married, n (%)	1,063 (53.15)	.19	611 (61.78)	.22
Have health insurance, n (%)	1,216 (60.80)	.08	635 (68.13)	.06
Employed, n (%)	1,596 (79.80)	.54	833 (84.23)	.26
Home owner, n (%)	1,445 (72.25)	<.001	779 (78.77)	.008
In good health, n (%)	1,288 (64.40)	.22	878 ( 89.32)	.76
Current smoker, n (%)	590 (29.50)	.06	292 (29.03)	.91
Former smoker, n (%)	523 (26.15)	.06	300 (29.82)	.91
Consumed alcohol in last year, n (%)	1,206 (61.25)	.22	628 ( 63.50)	.18
Any physical activity, n (%)	1,436 (72.90)	.67	858 (86.75)	.79
Walking (min/wk), mean (SD)	83.29 (245.56)	.76	82.45 (250.18)	.27
Physical activity (min/wk), mean (SD)	175.77 (386.90)	.38	181.34 (375.15)	.07

Abbreviations: BMI, body mass index; CES-D, Centers for Epidemiologic Studies–Depression.

a *P* values were assessed with analysis of variance for categorical variables and Pearson correlation coefficient for continuous variables and are for the association of participant characteristics with the (continuous) overall built environment score for the street segment of residence.

b Longitudinal sample; includes only those study subjects with 2 or more observations.

c CES-D score ≥16 indicates depression. CES-D scores can range from 0 to 60.

**Table 2:** Neighborhood Characteristics of Participants (N = 2,000) Included In Analyses Evaluating the Cross-Sectional and Longitudinal Associations of the Built Environment Around the Residence and Depression in a Rural Population, by Depression Status, Bogalusa Heart Study, 1998–2013

Variable	Cross Sectional			Longitudinal^a^		
	Depressed^b^ (n = 740)	Not Depressed (n = 1,260)	*P* Value^c^	Depressed^b ^(n = 568)	Not Depressed (n = 438)	*P *Value^c^
**Street segment of built environment^d^**						
All features, mean (SD)	10.55 (4.32)	10.49 (4.49)	.77	10.41 (4.17)	10.34 (4.43)	.78
Path, mean (SD)	2.31 (2.35)	2.20 (2.40)	.32	2.08 (2.22)	2.11 (2.37)	.83
Pedestrian safety features, mean (SD)	3.21 (1.81)	3.03 (1.81)	.03	3.02 (1.83)	2.94 (1.89)	.51
Aesthetics, mean (SD)	2.99 (1.47)	3.28 (1.60)	<.001	3.33 (1.55)	3.30 (1.57)	.76
Destinations^e^, mean (SD)	0.61 (1.22)	0.54 (1.14)	.20	2.92 (1.11)	2.94 (1.11)	.90
Physical security, mean (SD)	3.41 (1.45)	3.19 (1.32)	<.001	0.52 (1.10)	0.51 (1.13)	.73
Land use, mean (SD)	1.61 (0.90)	1.67 (0.95)	.17	1.67 (0.98)	1.66 (0.90)	.85
**Contextual variables**						
Population density, mean (SD)	556.75 (1,575.20)	603.25 (1,189.66)	.46	536.30 (1,774.73)	520.84 (839.53)	.86
Percentage poverty^f^, mean (SD)	30.06 (10.23)	27.20 (11.22)	<.001	28.75 (10.58)	28.41 (11.00)	.39

Abbreviation: CES-D, Centers for Epidemiologic Studies–Depression.

a Longitudinal sample; includes only those study subjects with 2 or more observations.

b CES-D score ≥16 indicates depression. CES-D scores can range from 0 to 60.

c *P* values for comparison of depressed and not-depressed subjects are from *t* tests.

d Built environment scores summarize features of street segments assessed with the Rural Active Living Assessment street segment audit tool, overall and within domains of features, with higher numeric scores indicating the presence of more features thought to promote physical activity. Scores have the following ranges: overall (2–29), path (0–9), pedestrian safety (0–10), aesthetics (0–6), destinations (0–11), physical security (0–6), and land use (0–5).

e Includes commercial and civic facilities.

f Neighborhood poverty was defined as the percentage of residents in a census tract living below the federal poverty level.

Depressed subjects in the cross-sectional sample lived on street segments with lower aesthetics (*P* < .001) and higher physical security (*P* < .001) scores than nondepressed subjects (Table 2). Depressed subjects in the cross-sectional sample lived in census tracts with higher neighborhood poverty (*P* < .001) than nondepressed subjects. We observed no significant differences between street segment scores or neighborhood contextual variables between depressed and nondepressed subjects included in the longitudinal sample.

Significant associations were observed for aesthetics and physical security built environment scales on the street segment of residence (Table 3). For each 1-point higher aesthetics score on the street segment of residence, the prevalence of depression was 4% lower (PRR = 0.96, 95% CI, 0.92–1.00), and while similar magnitude associations were observed in larger neighborhood buffers, these were not significant. For each 1-point higher physical security score on the street segment of residence, the prevalence of depression was 7% higher (PRR = 1.07, 95% CI, 1.02–1.11).

**Table 3:** Association of Neighborhood Built Environment Scores^a^ With Prevalence and Incidence of Depression Among Participants (N = 2,000), Bogalusa Heart Study, 1998–2013

Built Environment Score	Buffer Around Residence Unit				
	0.00 mi	0.25 mi	0.50 mi	1.00 mi	1.50 mi
**Cross-sectional, prevalence rate ratio (95% CI)**					
Overall	1.01 (0.99–1.02)	1.01 (0.99–1.03)	1.01 (0.99–1.03)	1.00 (0.98–1.03)	1.00 (0.98–1.02)
Path	1.01 (0.98–1.04)	1.02 (0.99–1.05)	1.02 (0.98–1.05)	1.01 (0.97–1.05)	1.01 (0.97–1.06)
Pedestrian safety	1.03 (1.00–1.06)	1.03 (0.99–1.08)	1.04 (0.98–1.09)	1.03 (0.97–1.09)	1.02 (0.96–1.08)
Aesthetics	0.96 (0.92–1.00)	0.97 (0.92–1.02)	0.97 (0.91–1.02)	0.96 (0.90–1.02)	0.95 (0.88–1.02)
Destinations^b^	1.04 (0.99–1.10)	1.04 (0.97–1.10)	1.04 (0.97–1.12)	1.02 (0.94–1.12)	1.02 (0.93–1.13)
Physical security	1.07 (1.02–1.11)	0.97 (0.84–1.12)	1.02 (0.87–1.21)	1.03 (0.86–1.24)	1.00 (0.82–1.22)
Land use	0.98 (0.92–1.04)	0.97 (0.89–1.05)	0.95 (0.86–1.05)	0.92 (0.83–1.03)	0.95 (0.85–1.06)
**Longitudinal, risk ratio (95% CI)**					
Overall	1.00 (0.99–1.01)	1.01 (0.99–1.02)	1.01 (0.99–1.03)	1.01 (0.98–1.03)	1.00 (0.98–1.03)
Path	1.00 (0.97–1.02)	1.00 (0.97–1.03)	1.01 (0.97–1.04)	1.00 (0.96–1.04)	0.99 (0.95–1.04)
Pedestrian safety	1.01 (0.98–1.04)	1.00 (0.97–1.04)	1.01 (0.97–1.05)	1.00 (0.96–1.04)	1.00 (0.95–1.05)
Aesthetics	1.00 (0.97–1.04)	1.01 (0.97–1.06)	1.02 (0.97–1.07)	1.02 (0.97–1.08)	1.02 (0.96–1.08)
Destinations^b^	1.01 (0.97–1.06)	1.06 (1.00–1.13)	1.07 (1.01–1.14)	1.08 (1.01–1.15)	1.07 (1.00–1.15)
Physical security	1.00 (0.96–1.05)	1.11 (0.96–1.28)	1.15 (0.97–1.36)	1.13 (0.94–1.37)	1.10 (0.90–1.35)
Land use	1.00 (0.95–1.06)	0.99 (0.92–1.05)	0.99 (0.93–1.07)	0.97 (0.90–1.05)	0.96 (0.88–1.05)

a Built environment scores summarize features of street segments assessed with the Rural Active Living Assessment street segment audit tool, overall and within domains of features, with higher numeric scores indicating the presence of more features thought to promote physical activity. Measures of association represent the relative prevalence or risk of depression associated with a 1-point increase in the specified built environment score. Scores have the following ranges: overall (2–29), path (0–9) pedestrian safety (0–10), aesthetics (0–6), destinations (0–11), physical security (0–6), and land use (0­5).

b Includes commercial and civic facilities.

Significant associations were observed between higher destination scores and increased risk of depression. For each 1-point higher destination score, the risk of depression was 1.06 (95% CI, 1.00–1.13), 1.07 (95% CI, 1.01–1.14), 1.08 (95% CI, 1.01–1.15), and 1.07 (95% CI, 1.00–1.15) times higher in buffers of radii of 0.25, 0.50, 1.00, and 1.50 miles, respectively (Table 3). No significant associations of built environment scales with depression were observed for the overall built environment, paths, pedestrian safety, aesthetics, physical security, and land use in longitudinal analyses.

Significant effect modification by neighborhood poverty was identified for the pedestrian safety features scale in 0.25-mile, 0.50-mile, 1.00-mile, and 1.50-mile buffers in cross-sectional Poisson regression models, with 1-point higher pedestrian safety score associated with significant increased prevalence of depression in high-poverty neighborhoods but not low-poverty neighborhoods (Figure). Neighborhood poverty significantly modified the relationship between the destination scale and depression in longitudinal analyses, with 1-point higher destination scores in buffer radii of 0.25 mile, 0.50 mile, 1.00 mile, and 1.50 miles associated with higher risk of depression in high-poverty but not low-poverty neighborhoods. Associations between built environment scales and depression were not significantly modified by population density, though a 1-point higher aesthetic score was associated with significantly lower prevalence of depression in buffer radii of 0, 0.25 mile, and 0.50 of a mile in high-density neighborhoods but not in low-density neighborhoods (Figure).

**Figure:**
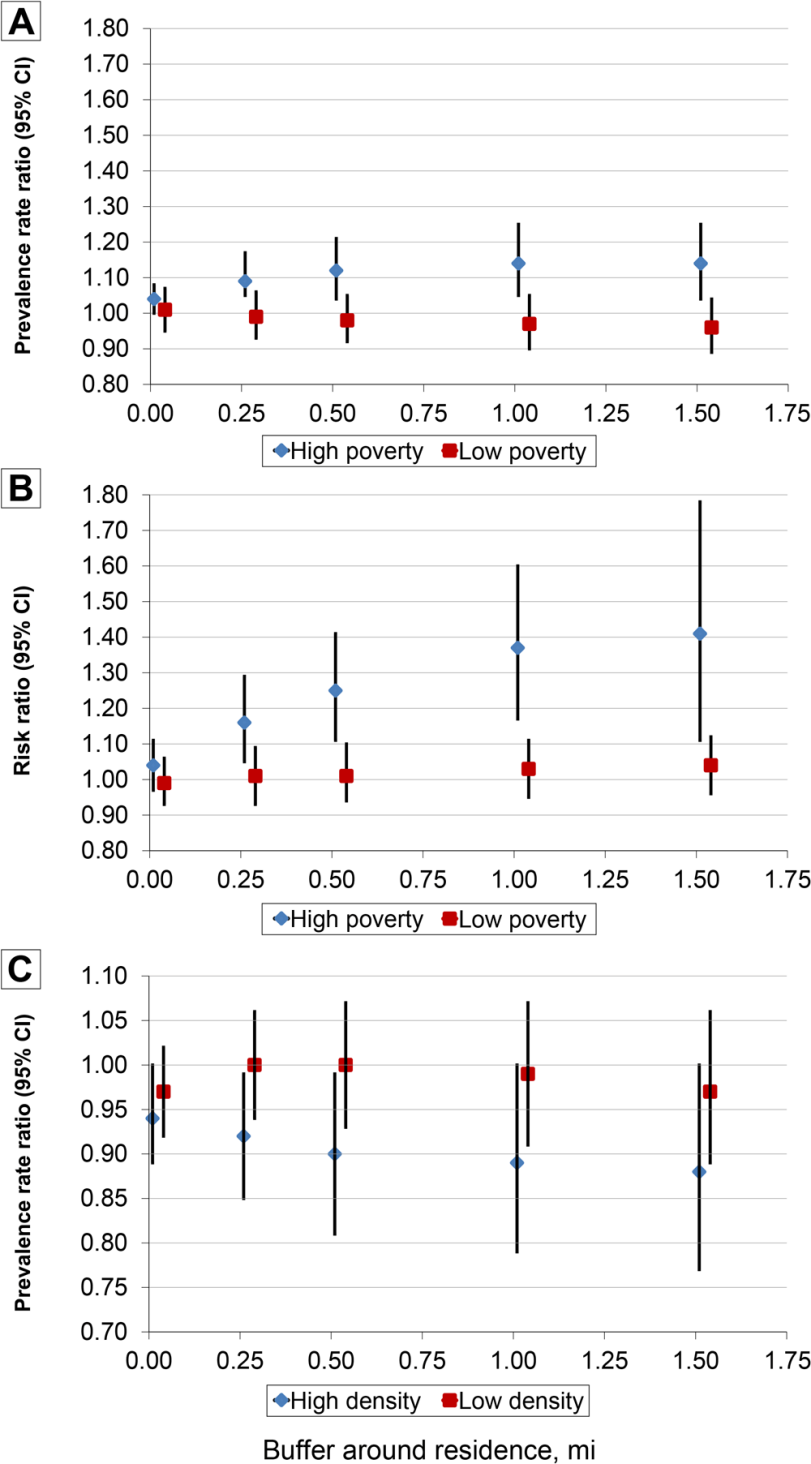
Association between built environment scores in buffers around residence and incident and prevalent depression among participants (N = 2,000) in the Bogalusa Heart Study, 1998–2013. High poverty is defined as ≥28.3% of residents (of a census tract) living below the federal poverty level; low poverty is defined as <28.3% of residents (of a census tract) living below the federal poverty level. High density is defined as ≥586 residents (of a census tract) per square mile of area; low density is defined as <586 residents (of a census tract) per square mile of area. Graph A shows prevalence rate ratio (PRR) for a 1-point increase in pedestrian safety score. Graph B shows the risk ratio (RR) for a 1-point increase in destination score, and graph C shows the PRR for a 1-point increase in aesthetics score.

**Table:** 

A. Buffer Radius, mi	High Poverty, PRR (95% CI)	Low Poverty, PRR (95% CI)
0.00	1.04 (1.00 to 1.08)	1.01 (0.95 to 1.07)
0.25	1.09 (1.01 to 1.17)	0.99 (0.93 to 1.06)
0.50	1.12 (1.04 to 1.21)	0.98 (0.92 to 1.05)
1.00	1.14 (1.05 to 1.25)	0.97 (0.90 to 1.05)
1.50	1.14 (1.04 to 1.25)	0.96 (0.89 to 1.04)
**B. Buffer Radius, mi**	**High Poverty, RR (95% CI)**	**Low Poverty, RR (95% CI)**
0.00	1.04 (0.97 to 1.11)	0.99 (0.93 to 1.06)
0.25	1.16 (1.05 to 1.29)	1.01 (0.93 to 1.09)
0.50	1.25 (1.11 to 1.41)	1.01 (0.94 to 1.10)
1.00	1.37 (1.17 to 1.60)	1.03 (0.95 to 1.11)
1.50	1.41 (1.11 to 1.78)	1.04 (0.96 to 1.12)
**C. Buffer Radius, mi**	**High Density, PRR (95% CI)**	**Low Density, PRR (95% CI)**
0.00	0.94 (0.89 to 1.00)	0.97 (0.92 to 1.02)
0.25	0.92 (0.85 to 0.99)	1.00 (0.94 to 1.06)
0.50	0.90 (0.81 to 0.99)	1.00 (0.93 to 1.07)
1.00	0.89 (0.79 to 1.00)	0.99 (0.91 to 1.07)
1.50	0.88 (0.77 to 1.00)	0.97 (0.89 to 1.06)

Over an average of 2.55 assessments, we found significant associations between the 10-year rate of change in depressive symptom severity and scales for the overall built environment and for paths, pedestrian safety, aesthetics, physical security, and land use (Table 4). In a buffer of 1.50 miles around the residence, a 1-point–higher overall and path scores were associated with 0.17 and 0.40 CES-D points/10-years slower increase in depressive symptom severity, respectively. Each 1-point higher pedestrian safety score was associated with 0.35, 0.52 and 0.57 CES-D points/10-years slower increase in depressive symptom severity in 0.50 mile, 1.00 mile, and 1.50 mile buffers, respectively. Neighborhood aesthetics were significantly associated with more rapid increases in severity of depressive symptom in all buffer radius sizes, with each 1-point higher aesthetics score associated with a 0.76 CES-D-points/10-years faster increase in depressive symptom severity in a 1.50-mile buffer radius. On the street segment of residence, each 1-point higher physical security score was associated with a 0.44 CES-D point/10-years faster increase in depressive symptom severity. In a 1-mile buffer radius, each 1-point higher land use score was associated with a 0.69 CES-D-points/10-years faster increase in depressive symptom severity. Of the significant slope differences, the only one mediated by physical activity was the aesthetics score on the street segment of residence (−2.83% mediated by physical activity) (Table 5).

**Table 4:** Association Between Neighborhood Built Environment Scores and Change in CES-D^a^, Participants (N = 1,006)^a^, Bogalusa Heart Study, 1998–2013

Built Environment Score^c^	CES-D Slope Difference^b^ for 1-Point Increase in Built Environment Score									
	Buffer Around Residence Unit									
	0.00 mi		0.25 mi		0.50 mi		1.00 mi		1.50 mi	
	β (SE)	*P *Value	β (SE)	*P *Value	β (SE)	*P *Value	β (SE)	*P *Value	β (SE)	*P *Value
Overall	0.02 (0.05)	.67	0.00 (0.06)	.98	−0.03 (0.07)	.69	−0.11 (0.08)	.16	−0.17 (0.08)	.04
Path	−0.07 (0.09)	.40	−0.09 (0.11)	.41	−0.09 (0.12)	.46	−0.22 (0.14)	.13	−0.40 (0.16)	.01
Pedestrian safety	−0.06 (0.11)	.60	−0.20 (0.14)	.16	−0.35 (0.16)	.03	−0.52 (0.17)	<.01	−0.57 (0.19)	<.01
Aesthetics	0.26 (0.13)	.04	0.51 (0.16)	<.01	0.67 (0.19)	<.01	0.71 (0.23)	<.01	0.76 (0.26)	<.01
Destinations^d^	0.08 (0.16)	.64	0.17 (0.24)	.49	0.05 (0.27)	.86	−0.10 (0.33)	.76	−0.36 (0.41)	.38
Physical security	0.44 (0.14)	<.01	0.22 (0.53)	.69	0.57 (0.62)	.36	0.46 (0.74)	.54	0.54 (0.84)	.52
Land use	0.27 (0.22)	.21	0.47 (0.27)	.08	0.55 (0.30)	.07	0.69 (0.34)	.04	0.71 (0.38)	.06

Abbreviation: CES-D, Centers for Epidemiologic Studies–Depression.

a Longitudinal sample; includes only those study subjects with 2 or more observations.

b CES-D slope was expressed as the rate of change in depressive symptom severity per 10 years of follow-up (depressive symptom severity was assessed as a continuous CES-D score that can range from 0 to 60, with higher scores indicating more severe depressive symptoms).

c Built environment scores summarize features of street segments assessed with the Rural Active Living Assessment street segment audit tool, overall and within domains of features, with higher numeric scores indicating the presence of more features thought to promote physical activity. Associations represent the difference in the rate of change of depressive symptom severity over 10 years for a 1-point increase in the specified built environment score. Scores have the following ranges: overall (2–29), path (0–9), pedestrian safety (0–10), aesthetics (0–6), destinations (0–11), physical security (0–6), and land use (0­5).

d Includes commercial and civic facilities.

**Table 5:** Percentage of the Observed Association of Neighborhood Built Environment Scores^a^ With CES-D Slope^b^ Mediated by Physical Activity, Participants (N = 1,006)^c^ Bogalusa Heart Study, 1998–2013

Built Environment	CES-D Slope Difference^b^, Percentage Mediation (95% CI) by Physical Activity				
	Buffer Around Residence Unit				
	0.00 mi	0.25 mi	0.50 mi	1.00 mi	1.50 mi
Overall	−5.92 (−20.12 to 4.33)	16.4 (−167.44 to 211.48)	−1.44 (8.23 to −12.09)	−0.06 (2.71 to −2.87)	0.23 (2.21 to −1.60)
Path	1.55 (7.87 to −3.70)	−0.51 (4.48 to −5.87)	0.50 (6.32 to −4.98)	0.40 (3.16 to −2.09)	0.35 (2.06 to −1.13)
Pedestrian safety	−0.84 (7.40 to −9.65)	0.40 (3.71 to −2.62)	0.24 (2.32 to −1.67)	0.35 (1.92 to −0.98)	0.55 (2.17 to −0.70)
Aesthetics	−2.83 (−6.59 to −0.29)	-0.31 (−1.83 to 1.01)	−0.13 (−1.38 to 1.04)	−0.15 (−1.53 to 1.12)	−0.18 (−1.59 to 1.10)
Destinations^d^	1.72 (−8.11 to 12.72)	0.79 (−5.58 to 7.70)	5.94 (−17.02 to 32.96)	−2.47 (10.41 to −17.04)	−0.92 (3.16 to −5.63)
Physical security	1.07 (−0.12 to 2.97)	−3.20 (−14.77 to 6.20)	−2.03 (−7.17 to 1.75)	−2.19 (−9.18 to 3.31)	−0.96 (−7.01 to 4.43)
Land use	2.48 (−0.66 to 7.21)	2.27 (−0.05 to 5.89)	2.29 (0.06 to 5.76)	1.83 (−0.05 to 4.78)	1.65 (−0.25 to 4.60)

Abbreviation: CES-D, Centers for Epidemiological Studies–Depression.

a Built environment scores summarize features of street segments assessed with the Rural Active Living Assessment street segment audit tool, overall and within domains of features, with higher numeric scores indicating the presence of more features thought to promote physical activity. Associations represent the percentage of the slope difference for a 1-point increase in the specified built environment score that is mediated by physical activity. Scores have the following ranges: overall (2–29), path (0–9), pedestrian safety (0–10), aesthetics (0–6), destinations (0–11), physical security (0–6), and land use (0­5).

b CES-D slope was expressed as the rate of change in depressive symptom severity per 10 years of follow-up (depressive symptom severity was assessed as a continuous CES-D score that can range from 0 to 60, with higher scores indicating more severe depressive symptoms).

c Longitudinal sample; includes only those study subjects with 2 or more observations.

d Includes commercial and civic facilities.

## Discussion

Our study identified significant associations between the built environment around a residence and depression and severity of depressive symptoms in the rural South. A more aesthetically pleasing street segment of residence was associated with a 4% lower prevalence of depression, and more security features (eg, window bars) were associated with a 7% higher prevalence of depression at the baseline visit. Each 1-point higher destination score within 0.25-mile, 0.50-mile, 1.00-mile, and 1.50-mile buffer radii around a residence was associated with a significant 6% to 8% higher risk of depression over an average of 10 years of follow-up. Effect modification by neighborhood poverty was identified, with significant associations observed exclusively in high-poverty neighborhoods between more pedestrian safety features and higher prevalence of baseline depression. Significant associations between more neighborhood destinations and higher risk of depression were identified exclusively in high poverty neighborhoods. No significant effect modification by neighborhood population density was observed.

Several studies have reported associations between the built environment and depressive symptoms (6,7). Among older adults, more walkable neighborhoods were associated with low cross-sectional odds of depression among men but not women (23). Neighborhood problems such as noise, vandalism, poor residential quality, incivilities (eg, trash on street), and heavy traffic were associated with more depressive symptoms at baseline but no changes in depressive symptoms over follow-up (24). A study among low-income Black and White residents of the southeastern US identified nonsignificantly higher odds of depression, with those in neighborhoods with the highest walkability index having 6% higher odds of CES-D–defined depression compared with those in the lowest walkability index neighborhoods (25). Our study did not identify associations between the overall built environment score and depression in cross-sectional or longitudinal analyses; however, the association between higher destination scores and increased risk of depression in our study is in accord with the higher odds of depression in the prior study (25). Differences between studies in the way neighborhood walkability was determined (by GIS mapping or street segment audit), the way scores were developed, and population density may explain the absence of an association between the overall score and depression in our study. The association between a high physical security score on the street segment of residence and increased depression prevalence at baseline is likely due to an inverse association of these features with residents’ perception of safety. Inverse associations between objective and perceived neighborhood safety measures and depressive symptoms have been reported previously (24,26). In our study, living on a more aesthetically pleasing street segment was associated with lower prevalence of depression at baseline, in accord with prior reports that less aesthetically pleasing environments such as those with trash in the streets (24) or with less greenspace (27) are associated with greater depression.

Reasons for differences in associations between built environment scores and depression at baseline and over follow-up are unclear, but similar patterns have been reported previously (6,26). In a prior study high neighborhood and individual level safety measures were associated with low CES-D scores at baseline, but neither was significantly associated with changes in that score over a 10-year period (26). In our study, aesthetics and physical security scores for the street segment of residence were associated with decreased and increased prevalence of depression at baseline, respectively, but not over follow-up. The absence of longitudinal associations is consistent with the prior claim that changes to built environment exposures may be more important to incidence of depressive symptoms than static exposure (26).

We identified significant effect modification by the percentage of residents in a neighborhood with incomes below the FPL. This is in accord with a previous study that found significant associations between increased walkability and increased depression only in the most socioeconomically deprived neighborhoods (25). Similarly, in the present study, significant associations between higher pedestrian safety scores and increased depression prevalence at baseline were identified only in high-poverty neighborhoods. Significant associations between higher destination scores and increased depression risk over follow-up were also identified only in high-poverty neighborhoods in the present study. This may be due to the associations between high neighborhood socioeconomic disadvantage (and poverty), low neighborhood social capital, and high depressive symptom (7). In our study, at baseline, each 10% increase in the prevalence of poverty in a census tract was associated with 12% higher prevalence of depression. The stronger associations between the built environment and depression in these neighborhoods may be due to a sense of vulnerability resulting from psychological stress, to which financial concerns are a significant contributor (28).

The mechanisms that underlie the association between higher physical security scores and increased prevalence of depression, between higher aesthetic scores and decreased prevalence of depression, and between higher destination scores and increased risk of depression are unknown. The associations between built environment scores and the rate of change in CES-D scores were not mediated by physical activity in our study population. Previous research has suggested that chronic stress, and associated hypocortisolism, among residents in neighborhoods with more objective and perceived stress-inducing features may explain relationships between neighborhood social disadvantage and negative health outcomes (29). Another study identified alteration of resting-state neural oscillatory activity in the cerebellum as a mechanism that could explain associations between environmental factors and depression (30). A study of the built environment and perceived social support and psychological distress among residents identified associations of features that promote direct social interaction with increased perceived social support and potential benefits for mental health (31). Associations between scores for built environment features and depression found in our study may therefore be mediated by influences on perceived social support or chronic stress.

Our study has several strengths. The sample was a well-characterized rural population with longitudinal data on physical activity, the built environment, and depression, and analyses were adjusted for potential individual and contextual confounders. Depression was assessed with a validated instrument (13), and the built environment scales were both reliable and associated with physical activity. Analyses evaluated cross-sectional and longitudinal associations. The density of street segments included in built environment audits allowed the construction of scales for the built environment within buffers of various radii around the residence, which has been a limitation in much of the prior literature (6). Our study also has limitations. Observed associations were based on an observational study design, and causality could not be inferred. No measure of the perceived built environment was available, so we could not assess mediation of associations between the objectively assessed built environment and depression by residents’ perception of the built environment. Also, no measure of social support was included in our analysis. The uncertain geographic context problem, in which the true geographic context relevant to the health outcome being studied for participants is unknown, may have contributed to underestimation of the strengths of the observed associations. Most study participants lived in one rural parish in Louisiana, so generalizability of the results may be limited.

Our study contributes to prior findings of cross-sectional associations between built environment features thought to promote physical activity (ie, walkability) and increased prevalence of depression in the southern United States. Additionally, significant associations of more overall built environment features, more pedestrian safety features, more physical security features, and more destinations with greater depression only in high-poverty neighborhoods supports prior reports about the modifying influence of neighborhood socioeconomic status on the relationship between the built environment and depression. Relationships between built environment features thought to promote physical activity and negative mental health outcomes in low-socioeconomic–status neighborhoods may be due to relationships between these features and increased stressors among people who perceive themselves as marginalized (25,28). Built environment improvements tailored to neighborhood contexts and residents’ wants and needs may have more broadly positive effects on community health. Further research is needed to identify mechanisms underlying associations between the built environment and depression and to explain why neighborhood socioeconomic status modifies the relationship between the built environment and depression.
